# ASO Author Reflections: Recurrence Patterns and Survival Outcomes in Clinical Stage IIB/IIC Melanoma: Can We Stratify Patients for Consideration of Neoadjuvant Immunotherapy?

**DOI:** 10.1245/s10434-025-18373-8

**Published:** 2025-09-17

**Authors:** Mohammad Saad Farooq, Giorgos C. Karakousis

**Affiliations:** Division of Endocrine and Oncologic Surgery, Hospital of the University of Pennsylvania, Philadelphia, PA USA

## Past

Landmark clinical trials have demonstrated the benefit of adjuvant immunotherapy for stage IIB/IIC melanoma.^1,2^ More recently, there has been growing interest in evaluating the neoadjuvant approach in this patient population; however, prior studies assessing long-term clinicopathologic outcomes generally report data based on pathologic stage after sentinel lymph node microstaging, and these data are not available at the time when neoadjuvant therapy is being considered. We therefore aimed to characterize real-world recurrence patterns and survival specifically in clinical stage IIB/IIC melanoma to contextualize outcomes for patient selection for neoadjuvant immunotherapy and for comparison with future neoadjuvant trials.

## Present

In this current study, we retrospectively analyzed a cohort of 229 clinical stage IIB/IIC melanoma patients who were treated at the University of Pennsylvania from 2006 to 2019. We found an estimated 2-year recurrence-free survival (RFS) of 69%. Overall, 44% of patients experienced a recurrence, of whom 64% experienced it within 2 years of surgery. Additionally, the presence of ‘pre-surgical’ lymphovascular invasion significantly increased the risk of recurrence.^3^ These data provide a benchmark for comparison with future neoadjuvant trials and for stratification of patients who are at higher risk for recurrence prior to obtaining surgical data.

## Future

We recently presented preliminary data from the phase II clinical trial NCT03757689 evaluating single-dose neoadjuvant pembrolizumab in clinical stage IIB/IIC melanoma, finding a 2-year RFS rate of 84%.^4^ The 2-year RFS we reported for the neoadjuvant therapy-naïve cohort in our current study was 69%. Additionally, the currently ongoing clinical trial Neo Reni II (NCT05418972) evaluating neoadjuvant relatlimab plus nivolumab in clinical stage IIB/IIC melanoma will also provide further insights into novel treatment approaches for this patient population.^5^

Upcoming clinical and translational data from NCT03757689 with evaluation of on-treatment biospecimens may also provide information on the efficacy of neoadjuvant immunotherapy, particularly if biological signals can be correlated to clinical outcome measures such as recurrence and sentinel node metastasis. Future directions of study include assessment of immune markers of therapeutic response in systemic circulation and within the sentinel node, as current histopathologic measures of treatment efficacy used in stage III disease (i.e. pathologic response) are not as applicable to clinical stage IIB/IIC patients. Such signals may allow for personalization and optimization of selection for neoadjuvant/adjuvant immunotherapy.
